# The role of space availability and affiliation in shaping equine social distances and dynamics

**DOI:** 10.1038/s41598-025-92943-4

**Published:** 2025-03-25

**Authors:** Laura Torres Borda, Ulrike Auer, Florien Jenner

**Affiliations:** 1 Department for Small Animals and Horses, Centre for Equine Health and Research, Equine Surgery Unit, University of Veterinary Medicine Vienna, Veterinaerplatz 1, Vienna, 1210 Austria; 2 Department for Small Animals and Horses, Anaesthesiology and Perioperative Intensive Care Medicine Unit, University of Veterinary Medicine Vienna, Vienna, Austria

**Keywords:** Horse, Equine, Social behaviour, Social proximity, Population density, Space availability, Animal behaviour, Behavioural ecology

## Abstract

Horses confined to enclosures with limited opportunities for spatial avoidance and escape experience heightened social stress and conflict. While forced proximity can pose welfare risks, voluntary social proximity is considered a positive indicator of well-being. To investigate the effects of space availability on spatiotemporal dynamics, social network structure and agonistic interactions, this study used ultra-wideband sensors to continuously (1 Hz) measure interindividual distances in 34 horses across three groups under paddock and field conditions. Interindividual distances increased in larger enclosures (paddock: median 8.3 m, field: 19 m), but closest associates remained nearer to each other than other dyads (paddock: 3.6 m vs. 8.3 m, field: 13.6 m vs. 19 m) and spent more time within 3 m (paddock: 42.34% vs. 20.59%, field: 9.81% vs. 1.85%). However, horses did not disperse maximally in larger spaces, staying within range of even their least frequent associates, suggesting a threshold distance for group cohesion. Network analysis revealed higher closeness centrality in the paddock, indicating a more integrated social network. Agonistic interactions were more frequent between close associates, occurred more often in the paddock than the field, and correlated negatively with absolute interindividual distances. These findings highlight the complex interplay between space availability and social dynamics and their importance for equine welfare.

**Supplementary Information:**

The online version contains supplementary material available at 10.1038/s41598-025-92943-4.

## Introduction

Equine behaviour is deeply rooted in their evolutionary history as grazing herd animals. Adapted to a nomadic lifestyle, wild horses traverse distances up to 9 km daily within vast home ranges of up to 40 km²^1^. As gregarious animals, they live in small, cohesive groups of typically five to six individuals that can aggregate into larger herds^2–11^, with a low population density of 0.45 to 3.8 horses per km²^12,13^. The stable composition of (semi-)feral equine groups, characterized by enduring bonds and established dyadic interaction patterns, in conjunction with horses’ highly developed social cognition and social memory, minimizes the occurrence and intensity of agonistic interactions^11,14–21^. These agonistic interactions, 80% of which are ritualized and non-contact, primarily serve to reinforce prioritized access to limited resources and defend personal space^11,14–22^.

In contrast, domestic horses, subjected to frequent changes in social groupings and confined to small enclosures with limited opportunities for spatial avoidance, experience heightened social pressures compared to their feral counterparts^14,20–26^. Since equine social tolerance is influenced by available space, a reduced ability of subordinate individuals to escape or maintain the personal distance of dominant conspecifics increases stress and the risk of conflict^14,20–27^. Indeed, horses in small enclosures exhibit a higher number of aggressive interactions and behavioural transitions^20,24,26–30^. Research indicates that for group-housed horses, a minimum enclosure size of 342 m² per horse is necessary to mitigate stress and prevent injuries^30^. Adequate space allowance prevents forced proximity and enables horses to spread out, allowing them to choose which group members they affiliate with^14,31–36^.

Given that horses only tolerate close companions within their personal space of approximately 3 m (or 2 horse lengths), voluntary social proximity reflects the social bond with a preferred partner and has been validated as a reliable indicator of positive welfare in adult horses^36^. Consequently, the spatiotemporal aspects of social interactions, encompassing both the closeness and duration of interactions, serve as an important indicator of equine social relationships^32,35–38^. While affiliative interactions are characterized by close proximity of longer duration, agonistic interactions typically prompt immediate separation following an approach^16,22,23,39,40^. Thus, analysing spatial relationships and dynamics not only provides valuable insights into equine social behaviour but also helps optimize equine welfare by identifying affiliative partners, recognizing incompatible dyads and detecting potential cases of social isolation, enabling targeted interventions to enhance group cohesion.

This study investigates the impact of enclosure size on interindividual distances, proximity patterns and social networks using ultrawide-band sensors with the aim to quantify the relationship between space availability and equine social behaviour^41^. We hypothesized that (1) absolute interindividual distances increase with space availability; (2) relative interindividual distances (distance/ space available per horse) are greater in smaller enclosures than in larger ones; (3) close associates maintain proximity regardless of space availability; and (4) the incidence of agonistic behaviours correlates negatively with absolute interindividual distances.

## Materials and methods

### Horses and management conditions

Three groups of horses, composed of a total of thirty-four mixed-breed horses (26 mares, 8 geldings, Suppl. Table 1) aged 6 to 32 years (mean age 20.2 years) were included in this study. The horses were housed at an equine sanctuary, where they had daily turn-out in either paddocks or fields, with varying enclosure sizes across the different turn-out conditions and groups (Table 1). Water was available ad libitum, and all horses were fed a hay- or grass-based diet. In the stables, which were bedded with shavings, horses received hay twice daily, morning and afternoon. During paddock turn-out, they had unlimited access to hay via a feeder with 12 slots, while in field turn-out, horses grazed without additional hay.

**Table 1:** Turn-out conditions of the three horse groups during tracking, detailing the dimensions (length (m) X width (m)), surface area (m^2^) of the paddocks and fields, the number of horses present and space (m^2^) available per horse (surface/number of horses).

Group	Turn-out	Dimension	Area	Number of horses	Space per horse
A	Paddock	30 × 15	450	8	56.25
	Field	65 × 40	2682	9	298
B	Paddock	30 × 16	480	11	43.63
	Field	60 × 50	3152	10	315.2
C	Paddock	115 × 9.5	1093	14	78.07
	Field	115 × 60	7213	14	515.21

Group A consisted of 8 horses (four mares, four geldings; mean age 24.9 years) during paddock tracking, and 9 horses during field tracking, with the introduction of one mare (“*Suse*”) two months prior to field tracking. All horses were housed in individual box stalls and had daily turn-out for 6 h.

Group B was composed of 11 horses (seven mares, four geldings; mean age 19.4 years) during paddock and 10 horses during field tracking, as one gelding (“*Alboro*”) died due to old age 7 weeks before the field tracking. These horses were also housed in individual box stalls with daily turn-out for 9 h.

Group C consisted of 14 horses (all mares; mean age 18.5 years). Three horses, due to advanced age and the need for additional feeding and rest, were housed in individual box stalls and joined the group for daily turn-out of 6 h. The remaining 11 horses stayed continuously in the paddock with access to a run-in shelter. The paddock was opened to the field during spring and summer.

## UWB proximity measurements

An ultra-wideband (UWB) wireless real-time location system (RTLOC^®^), with a resolution of 1 measurement per second (1 Hz) and a measurement range of a minimum of 5 centimetres and a maximum of approximately 120 m, was employed in this study. This system, validated under field conditions for proximity tracking in horses^37^, measured interindividual distances ten times per second and stored the average of these measurements. UWB sensors (8 cm x 7 cm x 2 cm, 95 g) were attached to the horses’ halters (Suppl. Figure 1) for the entire tracking duration.

All groups were tracked twice, once in a paddock for 10 days and once in a field for 6 (group A) or 7 (group B and C) days. Before each tracking period, the group composition remained stable for at least two months. Horses were tracked throughout their turn-out time, resulting in a total tracking duration of 254 h (group A: 52 h, group B: 82 h, group C: 120 h) in the paddock and 152 h (group A: 36 h, group B: 70 h, group C: 46 h) in the field.

Prior to analysis, data were cleaned to remove erroneous measurements, which may occur secondary to poor connection between two sensors due to interference by another horse moving between two sensors or similar obstructions. Erroneous data were identified as measurements that exceeded the maximum theoretical distance between horses within an enclosure or were smaller than 5 centimetres (impossible due to sensor placement between the ears). Additionally, measurements showing no variation for over 10 s were discarded, as proximity measurements are inherently variable^37^. To eliminate the influence of forced proximity around the single hay feeder, all measurements where both horses were within 3 m of the feeder were excluded from the analysis. Interdyadic distance measurements, where only one horse was near the hayfeeder were retained. After cleaning the data, technical replicates (i.e., distance between horses A-B and B-A), which have established high inter-device correlation^37^, were averaged for further analysis.

## Interindividual distances

The absolute interindividual distances between all horse dyads were measured during two turn-out conditions (paddock and field). To assess the dispersion of horse groups relative to available space and the number of individuals within that space, the relative interindividual distance between all horse dyads was calculated by dividing the median absolute interindividual distances (measured in cm) by the space available per horse in the enclosure (calculated as the total enclosure area in m² divided by the number of horses in the enclosure)^41^. Drawing on research on chimpanzee space use^41^, relative distances were used to differentiate between forced proximity, characterized by low absolute but high relative interindividual distance and voluntary social proximity, indicated by small absolute and relative distances^36,42^. The effect of space availability on the interindividual distance between horses was analysed by fitting a linear mixed-effects model. The distance measurements between horses were included as dependent variables, predicted by the enclosure size as an independent variable (factor with two levels: field and paddock). The group and the horse identity were included as random intercept effects.

## Closest associates

The percentage of time two horses spent in close proximity (≤ 3 m) and at various extended distance ranges (3 m < distance ≤ 6 m, 6 m < distance ≤ 9 m, 9 m < distance ≤ 12 m) was calculated by dividing the total number of measurements recorded within each range by the total number of distance measurements (measurement frequency = 1 Hz, meaning 1 measurement = 1 s). To evaluate the impact of social preference on proximity behaviour and agonistic interactions, the conspecific with whom each horse spent the most and least time within a distance of 3 m was identified as the closest associate^43^ and least frequent associate, respectively.

The effect of space availability on the percentage of time two horses spent within a 3-meter distance (a recognized cut-off for social proximity^10,35,37^) was analysed using a linear mixed-effects model. The dependent variable was the percentage of time spent within 3 m, predicted by the enclosure size (field or paddock) as an independent variable. Group and horse identity were modelled as random intercept effects.

## Network behaviour

To examine the social structure among horses based on their interindividual distances, adjacency matrices were calculated, followed by hierarchical clustering, and network analysis. The mean distance between each dyad was calculated using data aggregated over the entire observation period for both turnout conditions. To quantitatively evaluate the network structure, several centrality metrics were computed separately for each condition and for each group using binary adjacency matrices derived from distance matrices. Degree centrality was calculated to determine the number of direct connections each horse had within the network, indicating its level of social connectivity. Betweenness centrality was used to measure the frequency with which a horse is on the shortest paths between other horses, highlighting its role as an intermediary facilitating communication across the network. Closeness centrality was computed to evaluate how close a horse was to all other horses, based on the average shortest path lengths, thus indicating its overall integration into the social network. Binary adjacency matrices were generated by first determining the median distance between nodes (horses), and then converting the original distance matrices into binary format, with connections established if distances were below the median threshold. The effect of the space availability on these network metrics was analysed using a linear mixed-effects model with closeness centrality as the dependent variable and enclosure as the independent variable (factor with two levels: field and paddock). Group and the horse identity were modelled as random intercept effects. In addition, the impact of group on network metrics was assessed using mixed-effects analysis.

To visually represent the social structure within each group, network graphs were constructed. The binary adjacency matrices were transformed into undirected, weighted graphs using the igraph package. The Kamada-Kaway layout algorithm was applied to these graphs to position the nodes (horses), so that their distances on the graph approximated their actual distances as defined by the adjacency matrices.

### Agonistic behaviours

Occurrences of agonistic approaches, high intensity retreats and low intensity retreats in both paddock and field settings, were identified based on the proximity and approach speed. The key criteria for identifying agonistic approach events were (1) a decrease in interindividual distance of at least 100 cm between two horses to an interdyadic distance lower than 200 cm within 3 s (2) a minimum approach speed of 85 cm per second^37^, (3) involvement of only two horses per event, (4) consistent direction of movement. High-intensity retreats were identified using the same criteria as agonistic approaches, but with an increase in interindividual distance of at least 100 cm within 3 s following an approach. Low-intensity retreats were similarly defined but without a speed requirement. The rates of these events per horse per hour under both conditions were computed using the formula “rate = number of events / (“number of horses per group” × “tracking duration”)”.

Spearman correlations were calculated to assess the initial associations between space per horse, herd size, absolute and relative median interindividual distance in paddock and field, and the different types of agonistic interactions. Following this, the association between space per horse, herd size, absolute and relative median interindividual distance in paddock and field and the different types of agonistic interactions was assessed using stepwise multiple linear regression analysis using agonistic approaches in the paddock and field as dependent variables.

The speed of approaches and retreats was calculated as speed = (distance[n] - distance[n-1]) / (time[n] - time[n-1]). Here, distance [n] denotes the distance recorded at time point n (time[n]), and distance[n-1] signifies the distance recorded at the preceding time point n-1 (time[n-1]). A threshold of 800 cm/sec was established as the maximum possible speed, based on existing literature^44,45^, the physical condition of the aged horses studied, and the size of the enclosures. Any speeds exceeding this threshold were considered measurement errors and excluded from analysis.

To assess the accuracy of using approach speed to identify agonistic interactions, a random sample of ten agonistic approaches, ten high-intensity retreats, and ten low-intensity retreats was analysed and compared with corresponding video recordings (Suppl. Video 1–3). Video recordings were captured using a GoPro HERO4 camera (1280 × 960p, 60fps), which was installed on-site, ensuring comprehensive coverage of the paddock. Video recordings and distance measurements were synchronized using timestamps to facilitate the comparison of behavioural sequences.

### Statistical analysis

Statistical analysis was carried out using R, v. 4.2.2^46^ and Graphpad Prism (version 9.5.1). Given that the distribution of distance measurements was non-Gaussian, as determined by the D’Agostino & Pearson test, nonparametric analysis methods were employed^47^, with an alpha level of 0.05 for statistical significance. Comparisons of means were conducted using the Fisher-Pitman permutation test via the R package “coin”^48^, with p-values calculated through Monte Carlo sampling (1000 iterations).

## Results

### Interindividual distances

**Fig. 1:**
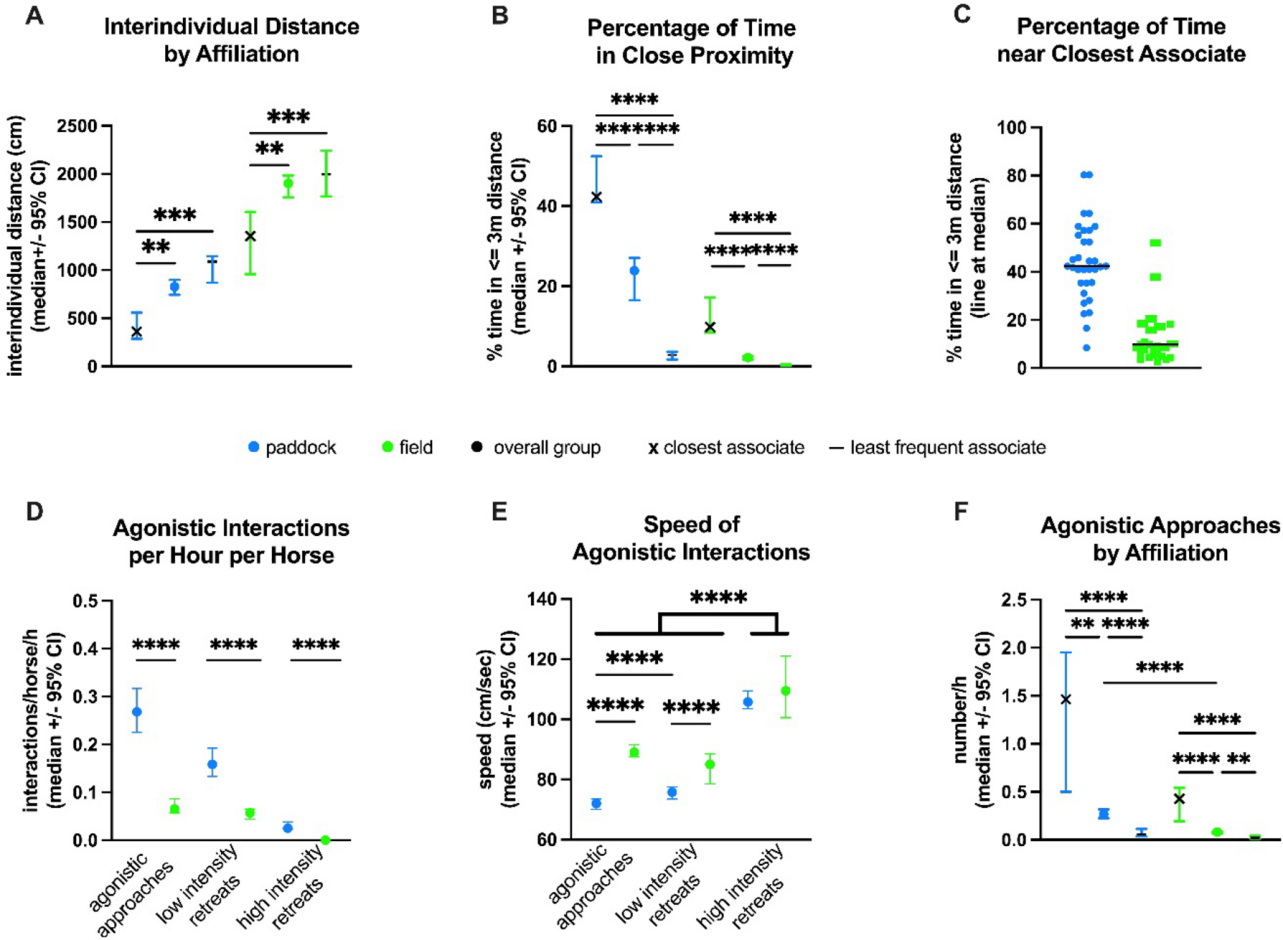
(**A**) Interindividual distances in the paddock and field based on affiliation levels. Closest associates (each horse’s primary companion, with whom it spent the most time within 3 m proximity) maintained significantly shorter distances than both the overall group and least frequent associates (for each horse the conspecific with whom it spent least time within ≤ 3 m). Distances for all groups were greater in the field than the paddock (*p* < 0.0001, significances are not displayed in the graph to enhance readability). (**B**) Percentage of time spent within ≤ 3 m proximity to group members in the paddock and field based on affiliation levels. Closest associates spent significantly more time together (*p* = 0.0002), and least frequent associates significantly less (*p* ≤ 0.0001), compared to the group overall. Across all affiliation levels, horses spent significantly less time in close proximity in the field compared to the paddock (*p* ≤ 0.0001). (**C**) Percentage of time closest associates spent in close proximity (≤ 3 m), showing individual variation across paddock (8.41 − 80.32%) and field (2.63 − 52%) conditions. (**D**) Frequency of agonistic approaches, low- and high-intensity retreats per hour per horse in the paddock and field. (**E**) Speed of agonistic approaches, low- and high-intensity retreats in the paddock versus the field. High intensity retreats were significantly faster than the other agonistic interactions (*p* ≤ 0.0001). Agonistic approaches and low intensity retreats were significantly faster in the field than the paddock (*p* ≤ 0.0001). In the paddock, agonistic approaches were significantly slower than retreats. (**F**) Frequency of agonistic approaches based on affiliation levels. Closest associates engaged in significantly more agonistic approaches (paddock: *p* = 0.0013, field: *p* ≤ 0.0001), whereas least frequent associates engaged in significantly fewer (paddock: *p* ≤ 0.0001, field: *p* = 0.0047) compared to the overall cohort in both environments.

The absolute interindividual distance in the paddock (median: 828 cm, 95% confidence interval (95% CI): 746–900) was significantly smaller (*p* < 0.001) than in the field (1903 cm, 95% CI: 1757–1985, Table 2), both overall (Z = 997.73) and within each group (Z = A: 575.44, B: 1317.3, C: 468.93). Even closest associates, while maintaining significantly closer proximity in the field than other dyads (*p* ≤ 0.0001), increased their interindividual distance from a median of 3.6 m in the paddock (95% CI: 2.9–5.6 m) to 13.6 m in the field (95% CI: 9.6–16.1 m). Notably, least frequent associates, while significantly farther apart than the group average in the paddock (median distance 10.9 m, 95% CI: 8.7–11.5 m vs. median 8.3 m, 95% CI: 7.5–9 m), did not disperse maximally during field turnout but remained at an interindividual distance similar to the overall group (median: 20 m, 95% CI: 17.7–22.4 m vs. median: 19 m, 95% CI: 17.6–19.9 m, Fig. 1A).

**Table 2:** Absolute and relative interindividual distances (median and 95% confidence interval (CI)) between horses according to space availability, across and within groups.

Group	Turn-out condition	Absolute interindividual distance (cm)		Relative interindividual distance (cm/m^2^)	
		Median	95% CI	Median	95% CI
Overall	Paddock	828	746–900	11	11–18
	Field	1903	1757–1985	4.3	3.7–6
A	Paddock	636	596–695	11.35	10.6–12.4
	Field	1089	956–1292	3.65	3.2–4.3
B	Paddock	858	772–1095	17.88	16.1–22.8
	Field	1732	1645–1815	6.04	5.7–6.3
C	Paddock	903	810–970	11.58	10.4–12.4
	Field	2153	2068–2278	4.18	4–4.4

In contrast to the absolute interindividual distance, the relative interindividual distance decreased significantly (*p* < 0.0001) with increasing space availability from a median of 12.2 cm/m^2^ (95% CI: 11.5–13.3 cm/m^2^) in the paddock to 4.5 cm/m^2^ (95% CI: 4.2–4.8 cm/m^2^) in the field (Table 2, Suppl. Figure 2A). Closest associates remained at a significantly closer relative distance than the group average in the paddock (median: 7 cm/m^2^, 95% CI: 4.9–7.8 cm/m^2^, *p* < 0.0001) and non-significantly greater proximity in the field (median: 3.1 cm/m^2^, 95% CI: 2.8–3.9 cm/m^2^, *p* = 0.143). Least frequent associates stayed at a non-significantly greater relative distance than the group average in both enclosures (paddock: median 14.9 cm/m^2^, 95% CI: 14.5–17.9 cm/m^2^, *p* = 0.197; field: 5.3 cm/m^2^, 95% CI: 4.7–5.9 cm/m^2^, *p* = 0.717).

Age and sex showed no significant effect on inter-individual distances (degrees of freedom: 168, R2: 0.06, Akaike Information Criterion: -122, *p* > 0.1).

### Closest associates

**Fig. 2:**
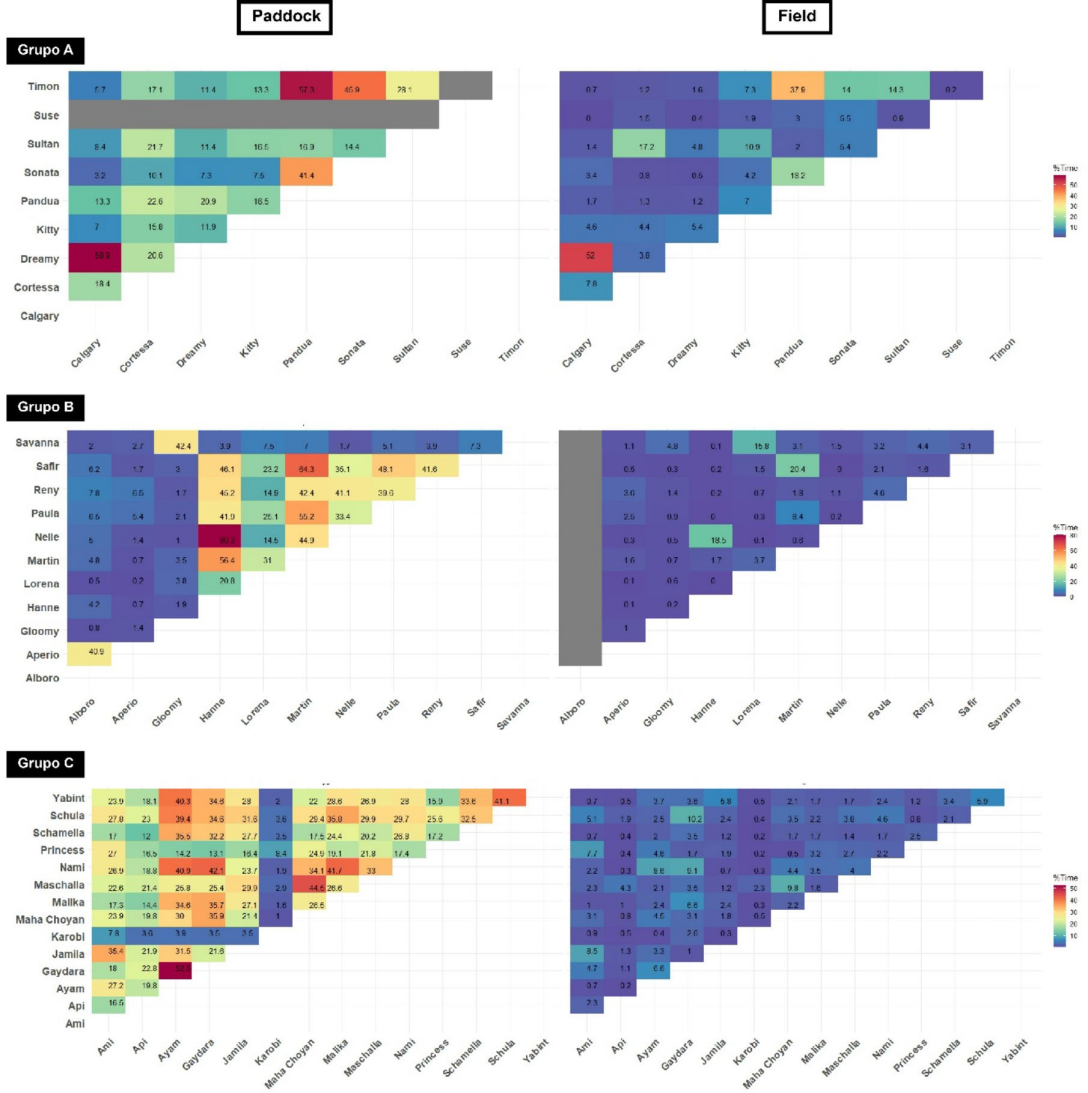
Percentage of time spent by horse dyads at less than 3 m from each other. All three groups in both conditions (field and paddock). Grey lines represent the absence of a horse during the tracking period.

Analysis of median distances between dyads and the percentage of time spent in close proximity (≤ 3 m) facilitated the identification of closest associates (Figs. 1B and C and 2, Suppl. Tables 2–4). Horses demonstrated a strong preference for their closest associates, spending significantly more time near them compared to other group members (*p* < 0.0001, Suppl. Figures 3–5).

While horses were only 23.87% of their time (median, 95% CI: 15.23–27.1%) in close proximity to other group members in the paddock, they remained near their closest associate for 42.34% of their time (median, 95% CI: 40.88–52.46%, range: 8.41–80.32%). However, the amount of time spent with the closest associate varied considerably among horses, with two individuals staying less than 20% of their time near their closest associate (*Karobi* (C group): 8.4%, *Kitty* (A group): 16.5%, Fig. 1C). Least frequent associates maintained proximity for only 2.87% of their time in the paddock.

Horses spent significantly less time at an interindividual distance ≤ 3 m from a conspecific in the field (median: 1.8%, 95% CI: 1.6–2.3%) than in the paddock (median: 19%, 95% CI: 17–23%, Z = 5.07, *p* < 0.001). However, time spent near their closest associate remained significantly higher than the group average at a median of 9.8% (95% CI: 8.44–17.19%), except for one horse (*Karobi*, C group) that spent only 2.63% of its time with its closest associate. Least frequent associates were only 0.25% of their time in the field near each other.

Proximity behaviour varied significantly between groups (*p* < 0.0001). Horses of group A spent least time near others in the paddock (median: 13.91%) compared to groups B (23.92%) and C (26.13%, *p* = 0.042), but spent most time in close proximity in the field (2.68% vs. 1.25% in B and 2.2% in C, *p* = 0.0007). For closest associates, group differences in the paddock were less pronounced (*p* = 0.083). However, in the field, A group horses spent significantly more time (18.25%, *p* = 0.0038) near their closest associate compared to those in the C group (median: 8.5%, B group: 15.38%).

### Network behaviour

**Fig. 3:**
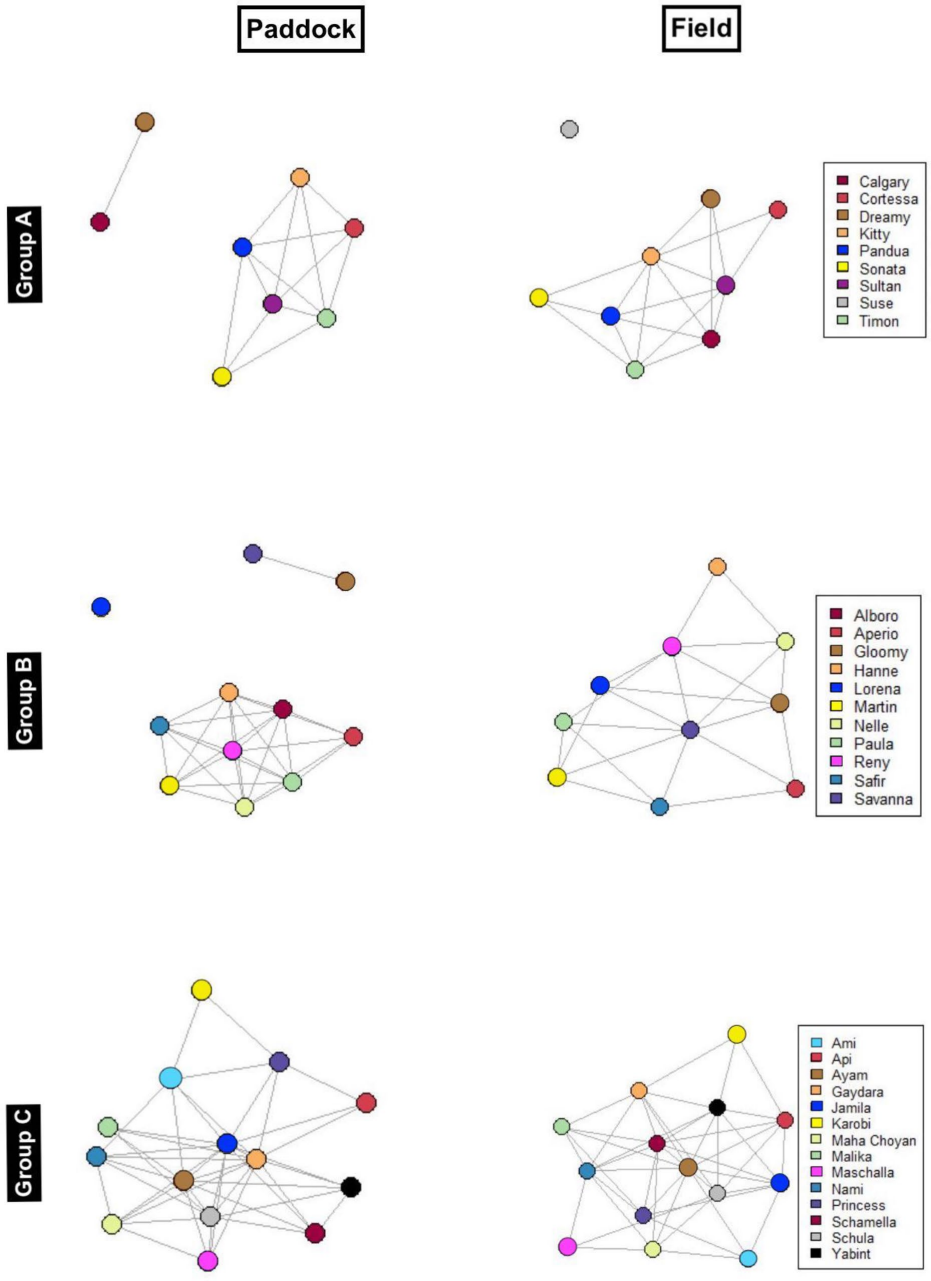
Network graphs (KK Visualisation) of the three horse groups (A, B and C) in both conditions (paddock and field). Nodes are coloured based on individual horse identities to maintain consistency across different conditions. Edge labels are added to represent weights, rounded to the nearest integer.

All three centrality metrics - degree centrality (*p* = 0.0061), betweenness centrality (*p* = 0.0137) and closeness centrality (*p* = 0.0078) - showed significant differences between groups (Table 3; Fig. 3). Degree and betweenness centrality were highest in group C and lowest in group A, whereas closeness centrality was highest in group A and lowest in group C.

**Table 3:** Network metrics according to group, condition and horse (degree centrality, betweenness centrality and closeness centrality).

Group	Turn-out	Horse ID	Degree Centrality	Betweenness Centrality	Closeness Centrality
A	Paddock	Calgary	1	0	1
		Cortessa	4	0	0.17
		Dreamy	1	0	1
		Kitty	4	0	0.17
		Pandua	5	0.67	0.2
		Sonata	3	0	0.14
		Sultan	5	0.67	0.2
		Timon	5	0.67	0.2
	Field	Calgary	5	0.67	0.11
		Cortessa	2	0	0.08
		Dreamy	3	0	0.09
		Kitty	7	5.33	0.14
		Pandua	5	0.67	0.11
		Sonata	3	0	0.09
		Sultan	6	2.67	0.13
		Suse	0	0	
		Timon	5	0.67	0.11
B	Paddock	Alboro	7	0.4	0.14
		Aperio	5	0	0.11
		Gloomy	1	0	1
		Hanne	7	0.4	0.14
		Lorena	0	0	
		Martin	6	0	0.13
		Nelle	7	0.4	0.14
		Paula	7	0.4	0.14
		Reny	7	0.4	0.14
		Safir	6	0	0.13
		Savanna	1	0	1
	Field	Aperio	3	0.5	0.06
		Gloomy	5	2.33	0.08
		Hanne	2	0	0.05
		Lorena	4	1.08	0.07
		Martin	4	0.83	0.07
		Nelle	4	2.08	0.07
		Paula	4	1.41	0.07
		Reny	6	6.58	0.08
		Safir	4	1	0.07
		Savanna	8	10.17	0.1
C	Paddock	Ami	6	7.4	0.05
		Api	3	0	0.04
		Ayam	10	5.92	0.06
		Gaydara	12	15.05	0.07
		Jamila	12	15.05	0.07
		Karobi	2	0	0.03
		Maha Choyan	6	0.2	0.05
		Malika	4	0	0.04
		Maschalla	5	0	0.05
		Nami	6	0.83	0.05
		Princess	5	4.6	0.05
		Schamella	5	0	0.05
		Schula	9	2.95	0.06
		Yabint	5	0	0.05
	Field	Ami	4	0.5	0.04
		Api	6	2.81	0.05
		Ayam	11	10.55	0.07
		Gaydara	7	4.94	0.05
		Jamila	7	3.42	0.05
		Karobi	3	0.2	0.04
		Maha Choyan	6	3.12	0.05
		Malika	5	0.34	0.05
		Maschalla	3	0.2	0.04
		Nami	8	6.43	0.06
		Princess	6	1.67	0.05
		Schamella	9	5.16	0.06
		Schula	7	5.74	0.05
		Yabint	8	4.93	0.06

Additionally, closeness centrality was significantly higher in the paddock compared to the field (Z=-2.46, *p* < 0.001), while degree centrality (Z=-0.057, *p* = 0.962) and betweenness centrality (Z = 1.177, *p* = 0.251) did not differ significantly between the two turnout conditions.

### Agonistic behaviours

The frequency of agonistic behaviours per hour per horse, including approaches and retreats of varying intensities, was significantly higher (*p* < 0.0001) in the paddocks compared to the fields (Fig. 1D-F, Suppl. Figure 2B-F). Groups in the most densely populated enclosures (B paddock, A paddock, A field, Table 1) exhibited the highest levels of agonistic interactions (Table 4, Suppl. Figure 2B-D).

**Table 4:** Distribution and speed (median and 95% confidence interval (CI)) of behavioural events (agonistic approaches, high-intensity retreats and low-intensity retreats) overall (O) and across different groups (A, B and C) and turn-out conditions (paddock (P) and field (F)).

		Agonistic approaches			High-intensity retreats			Low-intensity retreats		
Group	Turn-out	Number/ hour	Speed cm/sec (km/h)		Number/hour	Speed cm/sec (km/h)		Number/hour	Speed cm/sec (km/h)	
			Median	95% CI		Median	95% CI		Median	95% CI
O	O	22.54	75 (2.7)	73-76.5	2.26	106.5 (3.82)	104-109.5	12.85	76.88 (2.74)	75.5–78
O	P	30.96	72 (2.6)	70-73.5	3.09	106 (3.82)	104–110	17.33	75.8 (2.7)	73.5
	F	8.47	89 (3.2)	87.5–91	0.88	110 (3.96)	101–121	5.36	85 (3.06)	78.5–88.5
A	P	20.62	85.5 (3.06)	83–87	1.48	96.5 (3.46)	91–108	9.46	82 (2.95)	77.8–87
	F	15.72	88.6 (3.17)	84.2–92.5	1.94	108 (3.89)	97.5–122	9.67	86.2 (3.1)	79-90.9
B	P	48.46	78.5 (2.81)	76.3–80.5	4.94	102 (3.67)	99–105	27.89	73 (2.63)	70.5–75.5
	F	5.37	94.5 (3.38)	91-99.5	0.49	108 (3.89)	89.5–136	3.04	78.5 (2.81)	70.5–88
C	P	23.48	54.5 (1.94)	51-57.7	2.53	114 (4.1)	110–117	13.53	78 (2.81)	75-82.5
	F	7.5	82 (2.95)	70-88.5	0.63	110 (3.96)	98.5–146	5.5	86 (3.1)	71.6–93.5

Notably, closest associates engaged in significantly more agonistic interactions than the group overall or the least frequent associates (Fig. 1F, Suppl. Figure 2E, F). In the paddock, agonistic approaches between close associates (median: 1.5/h, 95% CI: 0.5–1.95) occurred 5.5 times more frequently than between dyads overall (0.27/h 95% CI: 0.23–0.32, *p* = 0.0013) and 25 times more than between least frequent associates (0.06/h 95% CI: 0.037–0.12, *p* < 0.0001). In the field, close associates also had the highest number of agonistic approaches compared to the group overall and least frequent associates (*p* < 0.0001, Fig. 1F). Similarly, low intensity retreats were most frequent between close associates in both paddock (*p* = 0.0019 vs. overall group, *p* < 0.0001 vs. least frequent associates) and field (*p* < 0.0001, Suppl. Figure 2E). High intensity retreats were also most frequent among close associates, though this reached significance only in the paddock (*p* = 0.01 vs. overall group, *p* < 0.0001 vs. least frequent associate, Suppl. Figure 2 F). All three agonistic interactions had a significant (*p* < 0.0001) negative correlation with absolute interindividual distances (agonistic approaches: *r*= -0.76, high-intensity retreats: *r*= -0.636, low-intensity retreats: *r*= -0.71) and very weak (*r* < 0.2, *p* < 0.05) positive correlation with relative interindividual distances.

Agonistic approaches were the most prevalent socionegative behaviour, with a total of 9151 (22.54/h) instances recorded, followed by low-intensity retreats (4937 instances, 12.16/h) and high-intensity retreats (919 instances, 2.26/h, Table 4). Agonistic approaches were 4.8 times as common in the paddocks (median: 2.6/h/horse, 95% CI: 1.7–4.4) than in the fields (median: 0.54/h/horse, 95% CI: 0.54–1.7). Group B had both the highest (4.41/h/horse in the paddock) and the lowest (0.54/h/horse in the field) frequencies of agonistic approaches.

The median speed of agonistic approaches was significantly higher (Z = 8.213, *p* < 0.001) in the field (89 cm/s (= 3.2 km/h), 95% CI: 87.5–91 cm/sec) compared to the paddock (72 cm/s (= 2.6 km/h), 95% CI: 70–73.5 cm/sec, Table 4).

For agonistic approaches in the paddock, regression analysis found a statistically significant linear relationship with median absolute and relative interindividual distance in the field, low intensity retreats, agonistic approaches and high intensity retreats in the field, enclosure size, herd size and percentage of time spent in proximity in either enclosure (degrees of freedom: 133, R^2^: 0.91, Akaike Information Criterion: -411.4, Table 5).

**Table 5:** Multiple linear regression model for factors influencing agonistic approaches in the paddock and field: detailing the estimate, standard error, the F-value with the degrees of freedom numerator (dfn) and degrees of freedom denominator (dfd), and the p-value.

Variable	Estimate	Standard error	F-Value (DFn, DFd)	*P*-Value
**Multiple linear regression model for agonistic approaches in the paddock**				
Intercept	0.1926	0.2882		0.0001
Median Distance (Paddock)	0.0005	0.0004	1.89 (1, 133)	0.172
Median Distance (Field)	0.0006	0.0002	5.83 (1, 133)	0.0171
Median Distance/Space (Paddock)	− 0.0289	0.022	1.72 (1, 133)	0.1915
Median Distance/Space (Field)	− 0.2977	0.0959	9.64 (1, 133)	0.0023
Agonistic approaches/h (Field)	1.273	0.3031	17.6 (1, 133)	0.0001
Low Intensity Retreats/h (Paddock)	1.313	0.1618	65.8 (1, 133)	0.0001
Low Intensity Retreats/Hour (Field)	− 2.122	0.5555	14.6 (1, 133)	0.0002
High Intensity Retreats/h (Paddock)	0.1997	0.7392	0.07 (1, 133)	0.7874
High Intensity Retreats/h (Field)	2.172	0.924	5.53 (1, 133)	0.0202
Paddock Area (m²)	− 0.0033	0.0009	13.2 (1, 133)	0.0004
Herd Size (Paddock)	0.2328	0.0796	8.56 (1, 133)	0.004
Percentage of Time < 3 m (Paddock)	0.0099	0.0029	11 (1, 133)	0.0012
Percentage of Time < 3 m (Field)	− 0.0182	0.00804	5.1 (1, 133)	0.0255
**Multiple linear regression model for agonistic approaches in the field**				
Intercept	− 0.1853	0.1240		0.0001
Median Distance (Paddock)	0.00005	0.0001	0.25 (1, 133)	0.6171
Median Distance (Field)	− 0.00006	0.00006	0.96 (1, 133)	0.3299
Median Distance/Space (Paddock)	− 0.001	0.006	0.03 (1, 133)	0.8715
Median Distance/Space (Field)	0.0494	0.0264	3.52 (1, 133)	0.0628
Agonistic approaches/h(Field)	0.0919	0.0219	17.6 (1, 133)	0.0001
Low Intensity Retreats/h (Paddock)	− 0.0171	0.0531	0.1 (1, 133)	0.7476
Low Intensity Retreats/h (Field)	1.667	0.0621	721(1, 133)	0.0001
High Intensity Retreats/h (Paddock)	− 0.326	0.1967	2.75 (1, 133)	0.0998
High Intensity Retreats/h (Field)	− 0.988	0.2385	17.2 (1, 133)	0.0001
Paddock Area (m²)	0.00004	0.00004	1.35 (1, 133)	0.2482
Herd Size (Paddock)	− 0.0194	0.0267	0.53 (1, 133)	0.4692
Percentage of Time < 3 m (Paddock)	− 0.0017	0.0008	4.3 (1, 133)	0.0401
Percentage of Time < 3 m (Field)	0.0188	0.0015	161 (1, 133)	0.0001

For agonistic approaches in the field, multiple linear regression analysis identified only low and high intensity retreats in the field, agonistic approaches in the paddock and the percentage of time spent in close proximity in either enclosure as significant factors (degrees of freedom: 133, R^2^: 0.98, Akaike Information Criterion: -797.7, Table 5),

“Low-intensity retreats” were the second most common behaviour, with 5216 (12.85/h) instances recorded. Like the other agonistic behaviours, these were more frequent in the paddock 76%, than in the field 24%. The median number of low-intensity retreats in the paddock per hour per horse was 1.18 (95% CI: 0.97–2.54) and in the field 0.39 (95% CI: 0.30–1.07, Table 4). The median speed for these retreats was significantly higher (Z = 6.248, *p* < 0.001) in the field (85 cm/s (= 3.06 km/h), 95% CI: 78.5–88.5 cm/sec) compared to the paddock (75.8 cm/s (= 2.7 km/h), 95% CI: 73.5–77.5 cm/sec, Table 4).

“High-intensity retreats,” though less common overall, displayed a similar pattern. Of the 919 (2.26/h) recorded instances, 78% occurred in the paddock and 22% in the field. The median number of high-intensity retreats in the paddock per hour per horse was 0.19 (95% CI: 0.18–0.45) and in the field 0.05 (95% CI: 0.05–0.22, Table 4). The median speed of these retreats was significantly higher (Z = 2.154, *p* = 0.0301) in the field (110 cm/s (= 3.96 km/h), 95% CI: 101–121 cm/sec) compared to the paddock (106 cm/s (= 3.82 km/h, 95% CI: 104–110 cm/sec, Table 4).

## Discussion

Spatiotemporal analysis revealed substantial variation in interindividual distances among horses, influenced by dyadic relationships and space availability. As anticipated, most horses stayed in close proximity primarily to particular companions while maintaining greater distances from other group members. In larger enclosures, where horses can exercise greater control over their interactions and express spatial preferences more freely, even close associates—while remaining significantly closer than other dyads—did not sustain the same level or duration of proximity observed in more confined settings. Conversely, even the least frequent associates did not maximize spatial separation or fully disperse in larger spaces; instead, they remained within space-dependent thresholds near the median distances of the group. The situative distancing observed in this study suggests that spacing behaviour is governed by a combination of individual social preferences, which shape close-range interactions, and the broader need to maintain group cohesion.

Group aggregation in animals can largely be explained by attraction-repulsion theory^49–61^, which posits that individuals too close to one another are driven apart by repulsive forces, while those beyond a certain distance are drawn together by attraction^49–61^. These dynamics result in an equilibrium distance at which repulsion shifts to attraction, often referred to as “personal space” or “repulsion zone”^49–61^. Although the attraction-repulsion theory provides a general framework for group-level spacing, it assumes uniform behaviour across individuals without accounting for dyad-specific spatial interactions. However, close-range proximity behaviours are more complex and less easily defined by deterministic models, as they are driven by personal preferences, with horses being drawn to some conspecifics while avoiding others^49–61^. Accordingly, these social behaviours may elicit asymmetric and individual-specific responses that are not necessarily reciprocal, highlighting the limitations of generalizing from group-level rules to individual-level social dynamics^49–61^.

Correspondingly, distinct distance thresholds seem to apply for different aspects of social behaviour, one for affiliative interactions, another marks the point beyond which a conspecific is no longer perceived as a threat, and a third defines the maximum distance that is not exceeded to ensure group cohesion. Distinguishing between these motivational systems and their associated distance thresholds is crucial, when investigating social spacing and interactions in horses. Traditionally, a distance of one to two body lengths (approximately 1.5–3 m) has been used as both, a benchmark for social proximity and affiliative behaviour in horses, and a threshold for group cohesion^33,62,63^. However, this study’s quantitative measurements challenge these fixed cut-offs, revealing that horses do not strictly adhere to these proximity thresholds, but instead display a wider range of interindividual distances. Consistent with our findings, previous drone-based studies have reported that a interindividual distance of three body lengths is typical for nearest neighbours in free-ranging horses, with the range spanning from one to more than 30 body lengths^64^, which is larger and more variable than the conventional proximity thresholds cited in the literature^33,62,63^. This suggests that rigid proximity thresholds may oversimplify the nuances of affiliative bonds and calls for a shift to a more nuanced understanding of equine social spacing that incorporates individual variability and environmental context.

While individual social preferences and personality may influence the thresholds for social interaction, the benchmark for maintaining group cohesion appears to be more consistent across individuals. However, both thresholds are subject to environmental factors, population density, and resource availability, leading to dynamic, context-specific boundaries for affiliation and cohesion. Therefore, instead of relying on fixed metric cut-offs, topological thresholds for social relationships and group cohesion may offer a more ecologically relevant approach to understanding social relationships and group cohesion^53,54,65–67^. Topological interaction parameters, which focus on the relative positioning of individuals within a group - such as their nearest neighbours or the relative interindividual distance per enclosure space and population density - rather than their absolute physical distances, capture the strength of social interactions regardless of group density or spatial constraints, making them well-suited for understanding social structure across varying environments^53,54,65–67^.

Horses in this study exhibited adaptive spacing, adjusting interindividual distances to enclosure size. While they spread out more in larger enclosures, they utilized proportionally more space when confined to smaller areas. This was evidenced by larger relative interindividual distances per available space in the paddocks compared to the fields, suggesting that proximity in smaller spaces was forced rather than voluntary^36,68^. This population density-dependent adaptive spacing is a fundamental ecological process also seen in other social species, such as caribou, to balance the costs and benefits of social living^69^. While sociality comes with certain disadvantages, such as increased social stress, more competition for resources and a greater risk of infectious disease transmission, group aggregation offers numerous benefits, including decreased predation, increased reproductive opportunities and enhanced foraging efficiency^60,70^. However, predation risk and feeding competition vary with an individual’s spatial position within the group, necessitating a trade-off between risk aversion and food availability^64,71^. Therefore, interindividual distances and positions within the group can be expected to change in response to predation risk and food dispersion. For instance, horses may increase the distance to their nearest neighbour when they are hungry and grazing but close the gap when they perceive a predation threat. Accordingly, the spatial configuration of an animal group reflects the cumulative responses of its individual members to the local environment, within the context of social relationships and responds adaptively to resource dispersion and threats^53^. Maintaining optimal distances is thus both a social strategy and an adaptive response to ecological pressures. Experience plays a key role in shaping these behaviours, as horses learn optimal spacing strategies to minimize conflict and maximize access to resources^14^. This adaptive spacing is ecologically important for prey species like horses, allowing them to remain within visual and auditory range of the group and balance risk avoidance with resource access^72^. However, domestic horses are frequently faced with restricted space, limiting their ability to adapt their interindividual distances, which causes social stress.

This study introduces an innovative method for coding social behaviours, specifically agonistic approaches and retreats, using quantitative data such as interindividual distance, proximity duration and speed to facilitate objective analysis of social interactions, moving beyond traditional observational techniques. Our results show a higher frequency of agonistic approaches and retreats in the restricted paddocks compared to the larger fields, as exemplified by group B having both the highest (4.41/h/horse in the paddock) and the lowest (0.54/h/horse in the field) frequencies of agonistic approaches, suggesting that space availability, rather than group composition, was the primary driver of these socionegative interactions.

This finding aligns with previous studies that observed more frequent agonistic interactions in smaller enclosures^24,28,73,74^. Confined spaces provide less room to establish and maintain personal space, leading to more frequent confrontations, higher stress and elevated cortisol levels^20,24,26–30,73–77^, highlighting the need to consider space and group density in managing horse welfare. In this study, only the field of group C provided space exceeding the recommendation of at least 342 m^2^ per horse, while the paddocks provided as little as 43.6 m^2^ per horse, leaving room for improvement, even though the frequency of agonistic approaches (2.6/h/horse) falls in the category of low aggressiveness defined as between 2 and 7 aggressive interactions per horse per hour in previous studies^20,22,24,28,30,73,74^. Notably, close associates engaged in more agonistic behaviours than non-affiliative dyads, suggesting that proximity, whether forced or voluntary, increases agonistic interactions^78^. Indeed, least frequent associates also had least frequent agonistic interactions.

Analysis of the interdyadic distances also revealed several horses that spent less time in proximity to their closest associate than the average of all dyads in a group and were outliers in the network analysis, indicating the lack of an affiliative partner and potential social isolation. These horses were involved in only few agonistic approaches per hour, indicating that lack of agonistic interactions may not be a good measure of group integration. Given the importance of sociopositive interactions for equine welfare, distance measurements and corresponding analyses of proximity, time spent near closest associates and network analysis may be a good tool to optimise group composition.

Whether horses were stabled in groups only during the day or for the entire day, there was no influence on interindividual distances, social cohesion, or the frequency of agonistic interactions. This consistency indicates that the observed behaviours are robust across different housing arrangements. These findings underscore the importance of adequate space in minimising stress and promoting harmonious social dynamics among horses^20,24,27,28,30,74,79^.

In this study, analogous to previous research^20,28,80^, no significant age- or sex-dependent differences in proximity or agonistic interactions were observed, but the older age of the study population may explain the relatively stable social structures and larger distances between individuals. Younger horses, by contrast, may show closer spacing due to a greater inclination to play and more dynamic interactions^20,81^. Previous studies support this, as older horses typically exhibit less physical activity and more established hierarchies, leading to less close physical contact^22^.

The older age average of the horses in this study contributes to its limitations, as it may not fully represent the potentially more active behaviours of younger populations. Additionally, the use of only one sensor per horse restricted our ability to determine the precise orientation of individuals toward each other during interactions, which may have provided deeper insights into spatial dynamics and social behaviour. Future studies should consider including younger populations and employing multiple sensors to further enhance our understanding of equine social interactions. Furthermore, while spatiotemporal measurements of proximity and interaction duration enable the broad categorization of social interactions as affiliative (prolonged proximity) or agonistic (immediate separation following approach), they currently lack the resolution to differentiate between specific behaviors within these categories. However these measurements hold promise for distinguishing affiliative and agonistic behaviors with overlapping characteristics, such as play-fighting and true fighting, which can be challenging to differentiate solely through observational methods. Both behaviours involve dynamic, close-range interactions but exhibit distinct spatiotemporal patterns. While play-fighting is typically marked by longer interactions, repeated reciprocal actions, alternating engagement and retreat between individuals, and sustained post-interaction proximity, true fights are generally shorter, involve unilateral approaches or aggressive pursuits, and result in definitive separation. Further research integrating proximity analysis with speed and dynamic movement data, alongside detailed video observations, will be crucial for developing robust and reliable metrics to characterize the spatiotemporal interaction patterns of specific behaviours.

## Conclusion

The findings of this study highlight the significant impact of spatial environment on equine social structures. Horses require sufficient space to align their interindividual distances with their social preferences and minimize both stress and the risk of injury. The observed flexibility in spatial arrangement suggests that interindividual distances for affiliation, avoidance and group cohesion are more dynamic and context-dependent than traditionally assumed. As a result, fixed metric thresholds may not fully capture the complex and fluid nature of equine spacing behaviour. Reevaluating these conventional cut-offs is essential to advancing our understanding of equine social dynamics and improving welfare assessments and management practices. Focusing on topological parameters, such as relative distances and social proximity, provides an ecologically more relevant and flexible framework for analysing equine social structure across different environments.

Analysing median inter-dyad distances and the percentage of time spent in close proximity helped identify affiliative pairs as well as horses at risk of social isolation. Given the importance of sociopositive interactions for equine welfare, these proximity patterns, along with network analysis, offer valuable insights for optimizing group composition.

As anticipated, smaller enclosures were associated with a higher frequency of agonistic interactions. However, interestingly, closest associates also engaged in more agonistic interactions than other dyads in the group, suggesting that both enforced proximity due to space limitations and voluntary proximity driven by social affinity may increase the likelihood of conflict. These findings illustrate the complexity of social dynamics in horses, where even close social bonds can give rise to tension, emphasizing the critical need for sufficient space to allow individual choice in social proximity or distancing, which is essential for animal welfare.

## Electronic supplementary material

Below is the link to the electronic supplementary material.

Supplementary Material 1

Supplementary Material 2

Supplementary Material 3

Supplementary Material 4

## Data Availability

The datasets generated and analysed during the current study are included in this published article (and its Supplementary Information files) or available from the corresponding author on reasonable request.
