# Supplementary material for: The role of space availability and affiliation in shaping equine social distances and dynamics
Source: Sci Rep. 2025 Mar 25;15:10273. doi: 10.1038/s41598-025-92943-4 (PMC11937558; doi:10.1038/s41598-025-92943-4)
Supplement: Supplementary file 1 — Supplementary Material 1 [file 41598_2025_92943_MOESM1_ESM.docx]

**Supplementary Material**

**Supplementary Figure Legends**


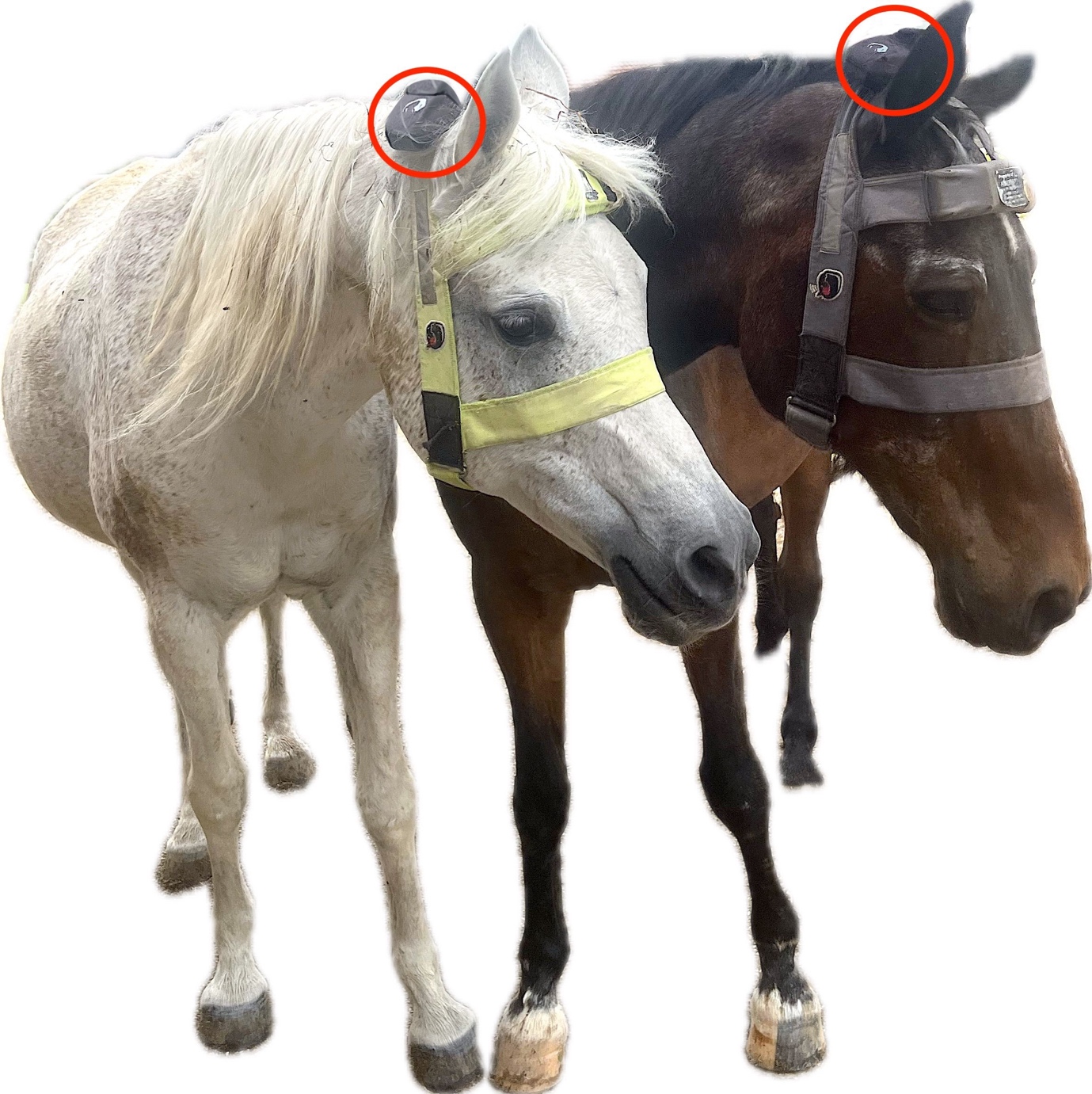


Supplementary Figure 1: Photograph of horses equipped with the UWB tag. The tag was attached to the horse's halter between the ears (indicated by the red circles).


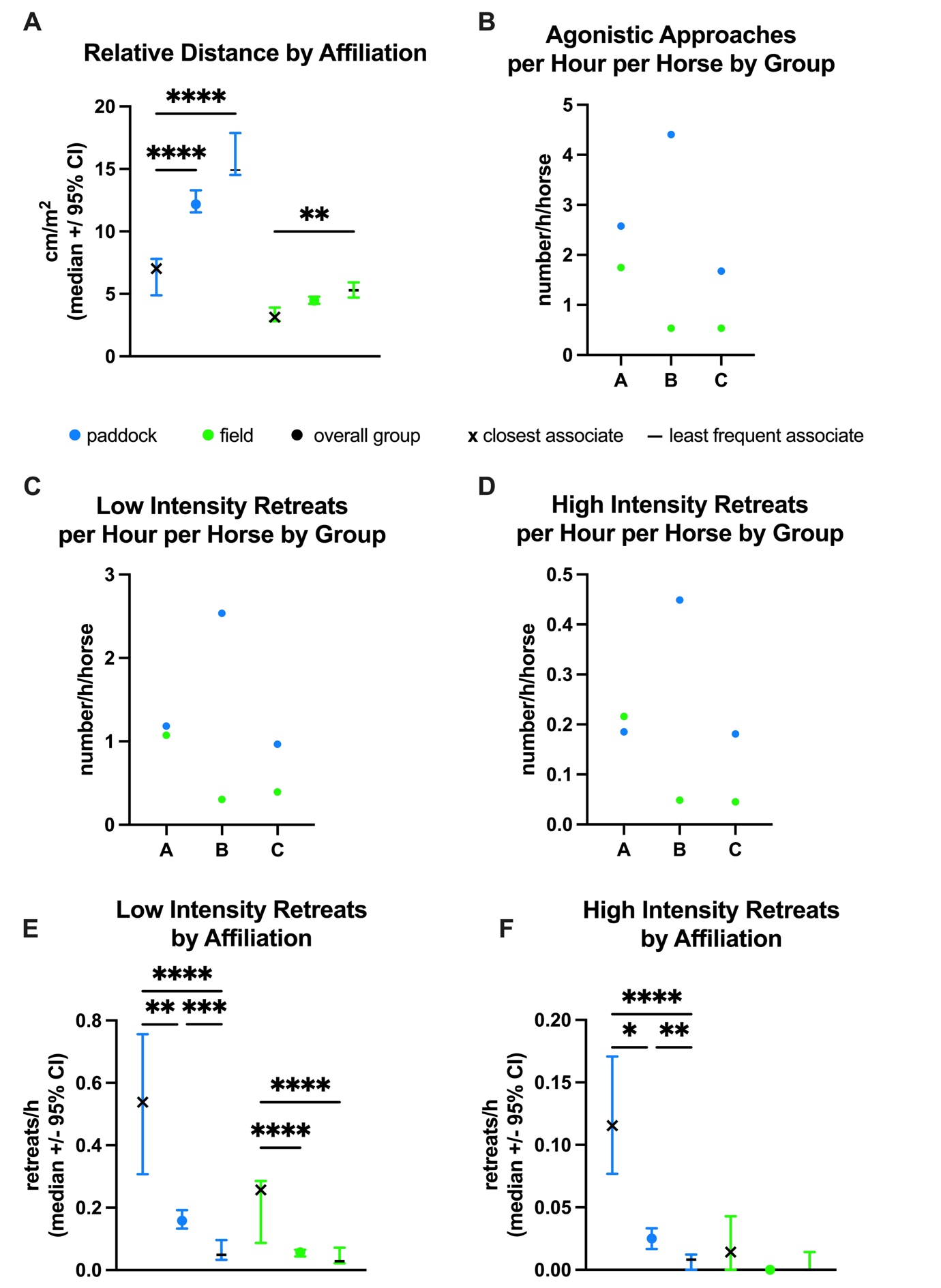


Supplementary Figure 2: (A) Comparison of the relative interindividual distance per available space (cm/m2) in paddock and field based on affiliation levels. Closest companions (each horse’s primary companion, with whom it spent the most time within 3m proximity) were (A) Comparison of the relative interindividual distance per available space (cm/m^2^) in paddock and field based on affiliation levels. Closest companions (each horse’s primary companion, with whom it spent the most time within 3m proximity) were significantly closer than both the overall group and least frequent associates (the horse with whom it spent least time within ≤3m). However, the distance between least frequent associates and the overall group in the field did not differ significantly. Regardless of affiliation level, all horses maintained significantly greater distances in the field compared to the paddock (p<0.0001, significances are not displayed in the graph to enhance readability).

(B) The graph illustrates the frequency of agonistic approaches per horse per hour in three groups of horses, both in a paddock and a field environment. As expected, a higher number of agonistic approaches were observed in the paddock compared to the field for all groups. The condition with the highest population density, group B during paddock turnout, exhibited the most frequent agonistic interactions.
(C) The graph illustrates the frequency of low-intensity retreats per horse per hour in three groups of horses, both in a paddock and a field environment. As expected, a higher number of retreats were observed in the paddock compared to the field for all groups. The condition with the highest population density, group B during paddock turnout, exhibited the most frequent low-intensity retreats.

(D) The graph illustrates the frequency of high-intensity retreats per horse per hour in three groups of horses, both in a paddock and a field environment. Except for group A, a higher number of retreats were observed in the paddock compared to the field. The condition with the highest population density, group B during paddock turnout, exhibited the most frequent high-intensity retreats.

(E) Number of low-intensity retreats per hour in the paddock and field, based on affiliation levels. Closest companions were more likely to engage in low-intensity retreats than the overall group, both in the paddock (p=0.0019) and the field (p≤0.0001). Least frequent associates, however, engaged in significantly fewer low-intensity retreats in the paddock (p=0.0003) but exhibited similar levels in the field.

(F) Number of high-intensity retreats per hour in the paddock and field, based on affiliation levels. In the paddock, closest companions exhibited a higher frequency of high-intensity retreats compared to the overall cohort (p=0.01), while least frequent associates engaged in significantly fewer high-intensity retreats (p≤0.0099).


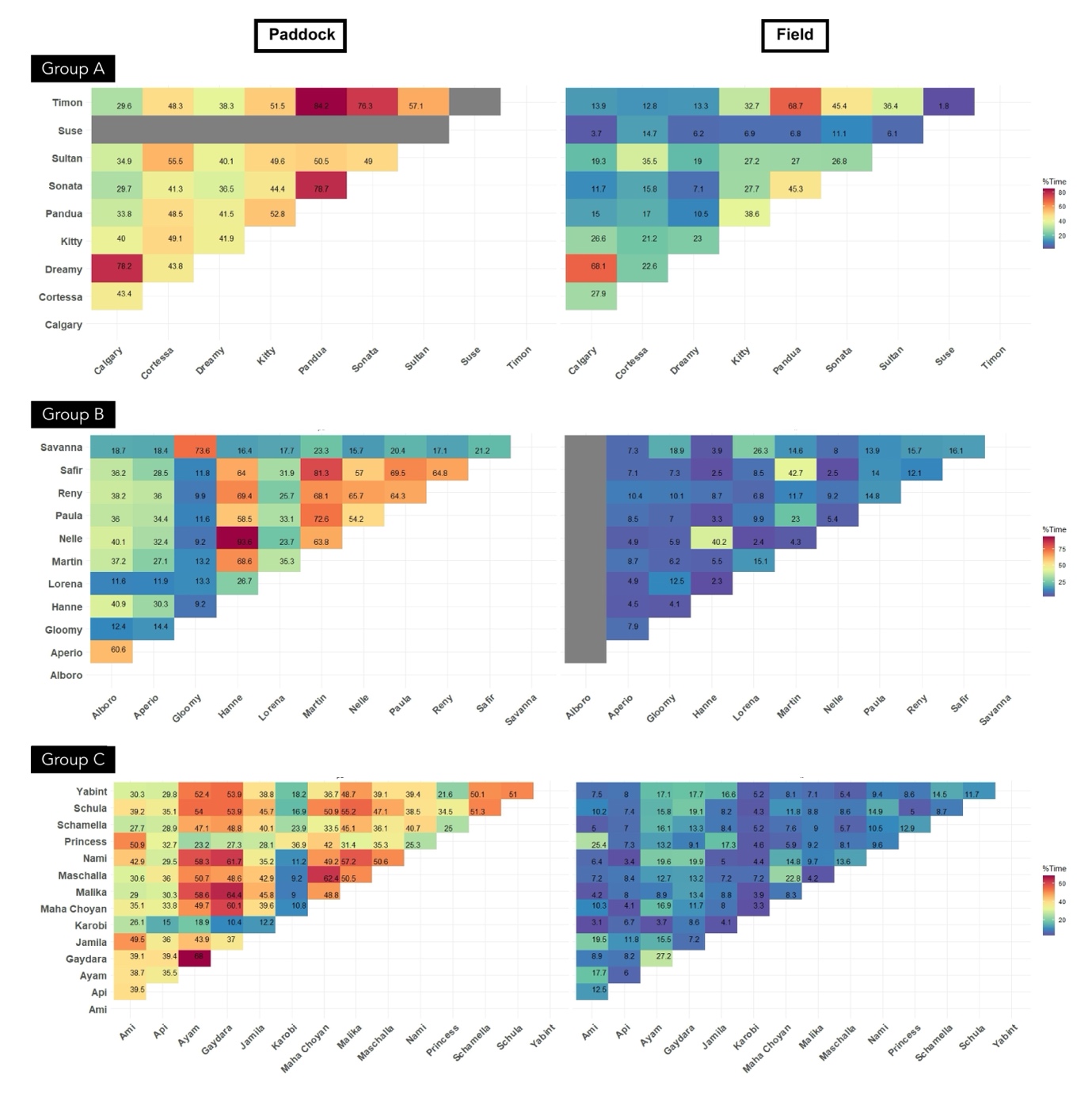


Supplementary Figure 3: Percentage of time spent by horse dyads of all three groups within 6m from each other in the field and the paddock. Grey lines represent the absence of a horse during the tracking period.


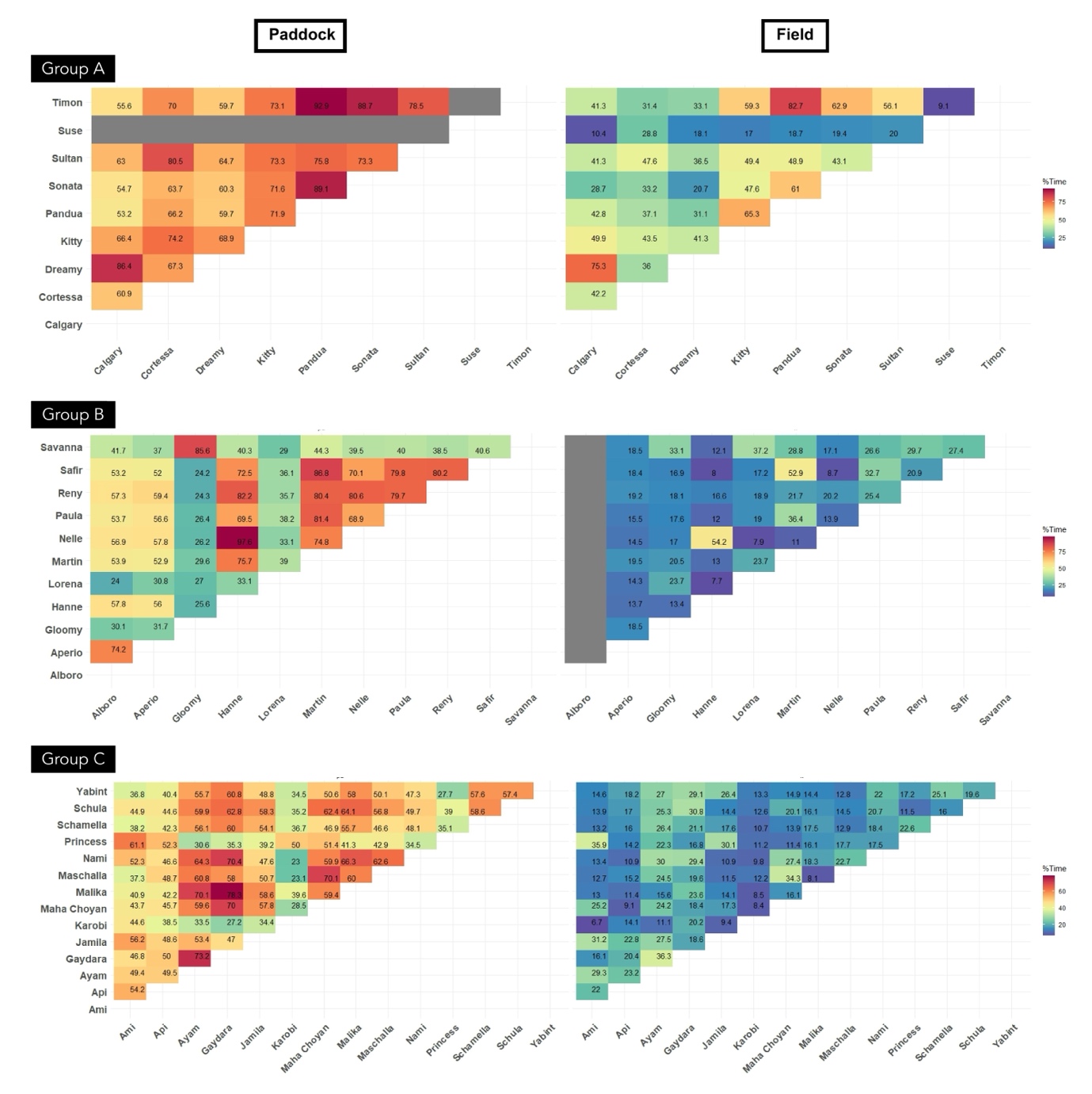


Supplementary Figure 4: Percentage of time spent by horse dyads of all three groups within 9m from each other in the field and the paddock. Grey lines represent the absence of a horse during the tracking period.


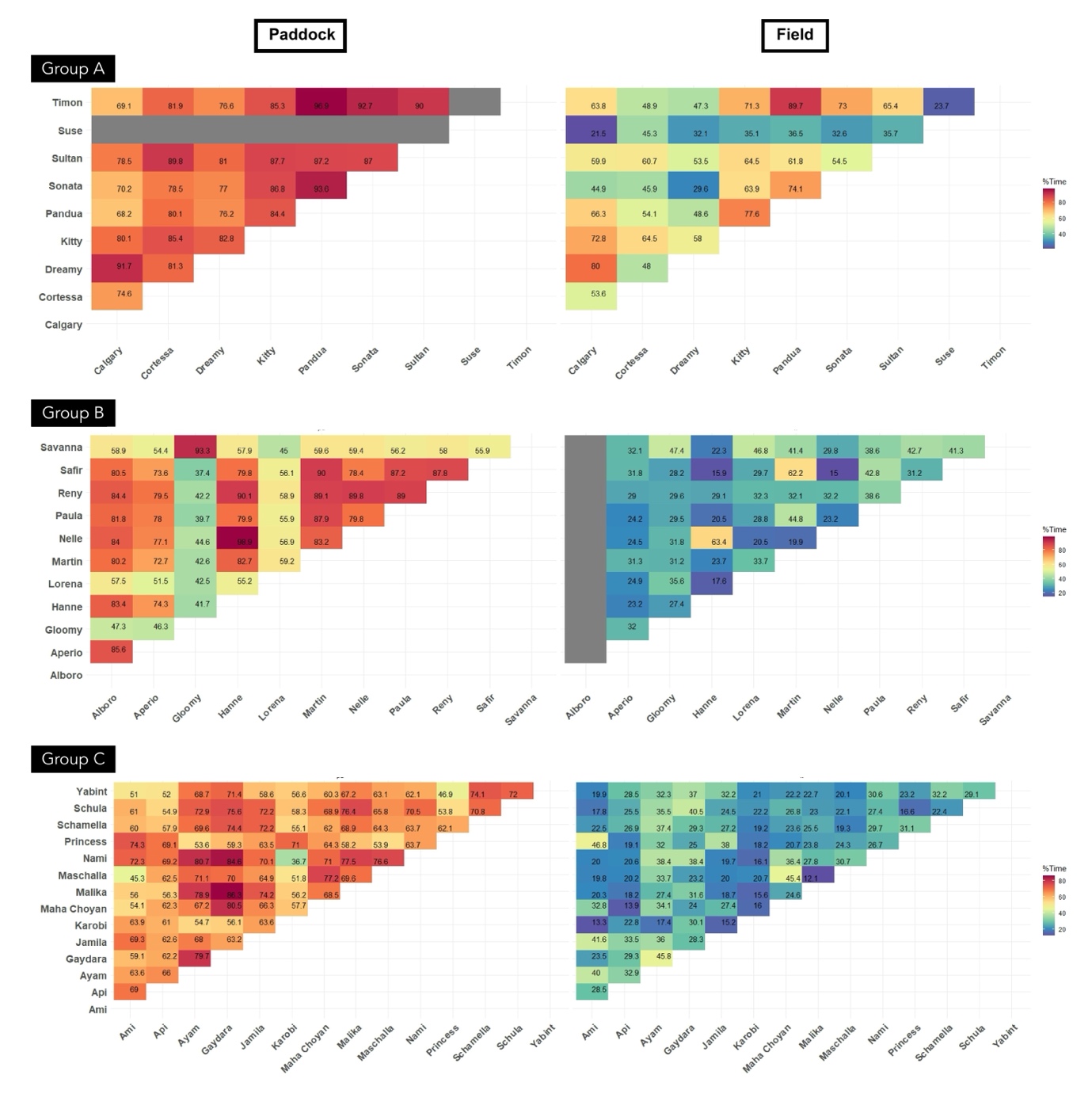


Supplementary Figure 5: Percentage of time spent by horse dyads of all three groups within 12m from each other in the field and the paddock. Grey lines represent the absence of a horse during the tracking period.

**Supplementary Video Legends**

Supplementary Video 1: Video sequence of an agonistic approach.

Supplementary Video 2: Video sequence of a low-intensity retreat.

Supplementary Video 3: Video sequence of a high-intensity retreat.

**Supplementary Tables and Table Legends**

Supplementary Table 1: List of all horses including group, age, sex and breed.

| **Group** | **Name** | **Age** | **Sex** | **Breed** |
| --- | --- | --- | --- | --- |
| Group A | Calgary | 25 | Gelding | Warmblood |
|  | Cortessa | 12 | Mare | Thoroughbred |
|  | Dreamy | 24 | Gelding | Tinker |
|  | Kitty | 29 | Mare | Warmblood |
|  | Pandua | 32 | Mare | Westfalian |
|  | Sonata | 30 | Mare | Warmblood |
|  | Sultan | 21 | Gelding | Friesian |
|  | Suse | 27 | Mare | Warmblood |
|  | Timon | 26 | Gelding | Warmblood |
| Group B | Alboro | 31 | Gelding | Standardbred- cross |
|  | Aperio | 27 | Gelding | Haflinger |
|  | Gloomy | 19 | Mare | Warmblood |
|  | Hanne | 20 | Mare | Romanian Warmblood |
|  | Lorena | 23 | Mare | Romanian Warmblood |
|  | Martin | 15 | Gelding | Romanian Warmblood |
|  | Nelle | 19 | Mare | Romanian Warmblood |
|  | Paula | 23 | Mare | Romanian Warmblood |
|  | Reny | 19 | Mare | Romanian Warmblood |
|  | Safir | 10 | Gelding | Haflinger |
|  | Savanna | 19 | Mare | Warmblood |
| Group C | Ami | 25 | Mare | Arabian |
|  | Api | 19 | Mare | Arabian |
|  | Ayam | 22 | Mare | Arabian |
|  | Gaydara | 26 | Mare | Arabian |
|  | Jamila | 6 | Mare | Arabian |
|  | Karobi | 21 | Mare | Arabian |
|  | Maha Choyan | 21 | Mare | Arabian |
|  | Malika | 15 | Mare | Arabian |
|  | Maschalla | 18 | Mare | Arabian |
|  | Nami | 24 | Mare | Arabian |
|  | Princess | 23 | Mare | Arabian |
|  | Schamella | 8 | Mare | Arabian |
|  | Schula | 19 | Mare | Arabian |
|  | Yabint | 12 | Mare | Arabian |

Supplementary Table 2 : Adjacency matrix of median distances (in cm) between horse pairs in group A in the paddock and field. Grey column represent the absence of a horse during the tracking period.

| **Paddock** | Calgary | Cortessa | Dreamy | Kitty | Pandua | Sonata | Sultan | Timon |  |
| --- | --- | --- | --- | --- | --- | --- | --- | --- | --- |
| Calgary | 0 | 831 | 423 | 810 | 941 | 972 | 841 | 959 |  |
| Cortessa |  | 0 | 741 | 700 | 738 | 807 | 626 | 731 |  |
| Dreamy |  |  | 0 | 764 | 816 | 866 | 795 | 851 |  |
| Kitty |  |  |  | 0 | 711 | 744 | 683 | 709 |  |
| Pandua |  |  |  |  | 0 | 464 | 689 | 358 |  |
| Sonata |  |  |  |  |  | 0 | 712 | 460 |  |
| Sultan |  |  |  |  |  |  | 0 | 604 |  |
| Timon |  |  |  |  |  |  |  | 0 |  |
| Suse |  |  |  |  |  |  |  |  |  |
|  | | | | | | | | | |
| **Field** | Calgary | Cortessa | Dreamy | Kitty | Pandua | Sonata | Sultan | Timon | Suse |
| Calgary | 0 | 1400 | 658 | 1048 | 1264 | 1572 | 1278 | 1321 | 1763 |
| Cortessa |  | 0 | 1475 | 1198 | 1507 | 1706 | 1139 | 1628 | 1456 |
| Dreamy |  |  | 0 | 1262 | 1610 | 1932 | 1384 | 1643 | 1642 |
| Kitty |  |  |  | 0 | 957 | 1223 | 1132 | 1053 | 1548 |
| Pandua |  |  |  |  | 0 | 1004 | 1239 | 552 | 1652 |
| Sonata |  |  |  |  |  | 0 | 1408 | 994 | 1753 |
| Sultan |  |  |  |  |  |  | 0 | 1154 | 1632 |
| Timon |  |  |  |  |  |  |  | 0 | 1896 |
| Suse |  |  |  |  |  |  |  |  | 0 |

Supplementary Table 3: Adjacency matrix of average distances (in cm) between horse pairs in group B in the paddock and field. Grey column represent the absence of a horse during the tracking period.

| **Paddock** | Alboro | Aperio | Gloomy | Hanne | Lorena | Martin | Nelle | Paula | Reny | Safir | Savanna |
| --- | --- | --- | --- | --- | --- | --- | --- | --- | --- | --- | --- |
| Alboro | 0 | 596 | 1422 | 805 | 1167 | 854 | 801 | 840 | 804 | 860 | 1259 |
| Aperio |  | 0 | 1441 | 906 | 1249 | 929 | 878 | 861 | 828 | 928 | 1299 |
| Gloomy |  |  | 0 | 1260 | 1325 | 1241 | 1246 | 1290 | 1270 | 1296 | 476 |
| Hanne |  |  |  | 0 | 1019 | 531 | 212 | 650 | 529 | 617 | 1088 |
| Lorena |  |  |  |  | 0 | 943 | 1034 | 1035 | 1031 | 1019 | 1225 |
| Martin |  |  |  |  |  | 0 | 593 | 479 | 549 | 416 | 1052 |
| Nelle |  |  |  |  |  |  | 0 | 685 | 542 | 678 | 1094 |
| Paula |  |  |  |  |  |  |  | 0 | 553 | 523 | 1099 |
| Reny |  |  |  |  |  |  |  |  | 0 | 555 | 1099 |
| Safir |  |  |  |  |  |  |  |  |  | 0 | 1091 |
| Savanna |  |  |  |  |  |  |  |  |  |  | 0 |
|  | | | | | | | | | | | |
| **Field** | Alboro | Gloomy | Hanne | Lorena | Martin | Nelle | Paula | Reny | Safir | Savanna |  |
| Alboro | 0 | 1829 | 2336 | 2030 | 2064 | 2099 | 2221 | 2105 | 1937 | 1786 |  |
| Gloomy |  | 0 | 2051 | 1873 | 2020 | 1885 | 2085 | 1894 | 2079 | 1511 |  |
| Hanne |  |  | 0 | 2242 | 2274 | 1183 | 2276 | 1895 | 2526 | 2140 |  |
| Lorena |  |  |  | 0 | 2007 | 2024 | 2093 | 1870 | 2166 | 1562 |  |
| Martin |  |  |  |  | 0 | 2196 | 1833 | 2041 | 1312 | 1708 |  |
| Nelle |  |  |  |  |  | 0 | 2168 | 1763 | 2351 | 1828 |  |
| Paula |  |  |  |  |  |  | 0 | 1783 | 1859 | 1779 |  |
| Reny |  |  |  |  |  |  |  | 0 | 2047 | 1599 |  |
| Safir |  |  |  |  |  |  |  |  | 0 | 1694 |  |

Supplementary Table 4: Adjacency matrix of average distances (in cm) between horse pairs in group C in the paddock and field.

| **Paddock** | Ami | Api | Ayam | Gaydara | Jamila | Karobi | Maha Choyan | Malika | Maschalla | Nami | Princess | Schamella | Schula | Yabint |
| --- | --- | --- | --- | --- | --- | --- | --- | --- | --- | --- | --- | --- | --- | --- |
| Ami | 0 | 1948 | 1758 | 1744 | 1227 | 1681 | 2184 | 2825 | 2857 | 1714 | 1421 | 2266 | 2268 | 2371 |
| Api |  | 0 | 1975 | 1732 | 1529 | 1872 | 2129 | 3041 | 1981 | 2310 | 1659 | 2251 | 2237 | 2590 |
| Ayam |  |  | 0 | 872 | 1297 | 2688 | 1347 | 1619 | 1554 | 1101 | 2341 | 1691 | 1150 | 1515 |
| Gaydara |  |  |  | 0 | 1282 | 2204 | 1294 | 978 | 1599 | 983 | 1707 | 1315 | 909 | 1259 |
| Jamila |  |  |  |  | 0 | 2059 | 1503 | 1699 | 1542 | 1463 | 1567 | 1328 | 1177 | 1459 |
| Karobi |  |  |  |  |  | 0 | 2634 | 2536 | 2983 | 3174 | 1461 | 2093 | 2459 | 2510 |
| Maha Choyan |  |  |  |  |  |  | 0 | 2438 | 1369 | 1378 | 1932 | 2244 | 1624 | 1918 |
| Malika |  |  |  |  |  |  |  | 0 | 2304 | 1760 | 3330 | 2099 | 1353 | 1977 |
| Maschalla |  |  |  |  |  |  |  |  | 0 | 1847 | 2164 | 2202 | 1616 | 1803 |
| Nami |  |  |  |  |  |  |  |  |  | 0 | 2599 | 2118 | 1568 | 2092 |
| Princess |  |  |  |  |  |  |  |  |  |  | 0 | 2108 | 2294 | 2589 |
| Schamella |  |  |  |  |  |  |  |  |  |  |  | 0 | 1437 | 1137 |
| Schula |  |  |  |  |  |  |  |  |  |  |  |  | 0 | 1293 |
| Yabint |  |  |  |  |  |  |  |  |  |  |  |  |  | 0 |
|  | | | | | | | | | | | | | | |
| **Field** | Ami | Api | Ayam | Gaydara | Jamila | Karobi | Maha Choyan | Malika | Maschalla | Nami | Princess | Schamella | Schula | Yabint |
| Ami | 0 | 2905 | 2208 | 3268 | 2335 | 3462 | 2661 | 2941 | 3734 | 3071 | 2197 | 2930 | 3182 | 3430 |
| Api |  | 0 | 2447 | 2750 | 2400 | 2728 | 3231 | 2743 | 2753 | 2949 | 3280 | 2515 | 2583 | 2666 |
| Ayam |  |  | 0 | 2164 | 2368 | 3309 | 2496 | 2532 | 2743 | 2164 | 2462 | 2303 | 2384 | 2647 |
| Gaydara |  |  |  | 0 | 2778 | 2679 | 2916 | 2657 | 2763 | 2184 | 2904 | 2652 | 2358 | 2470 |
| Jamila |  |  |  |  | 0 | 3337 | 2909 | 3095 | 3108 | 2936 | 2333 | 2459 | 2713 | 2505 |
| Karobi |  |  |  |  |  | 0 | 3285 | 2776 | 2799 | 3079 | 3605 | 2980 | 2831 | 2732 |
| Maha Choyan |  |  |  |  |  |  | 0 | 2811 | 2116 | 2022 | 2850 | 2543 | 2662 | 2845 |
| Malika |  |  |  |  |  |  |  | 0 | 3388 | 2429 | 2605 | 2413 | 2845 | 3050 |
| Maschalla |  |  |  |  |  |  |  |  | 0 | 2708 | 3538 | 3076 | 2710 | 3161 |
| Nami |  |  |  |  |  |  |  |  |  | 0 | 2404 | 2418 | 2766 | 2637 |
| Princess |  |  |  |  |  |  |  |  |  |  | 0 | 2247 | 3348 | 3110 |
| Schamella |  |  |  |  |  |  |  |  |  |  |  | 0 | 2734 | 2508 |
| Schula |  |  |  |  |  |  |  |  |  |  |  |  | 0 | 2638 |
| Yabint |  |  |  |  |  |  |  |  |  |  |  |  |  | 0 |
